# Discriminating Prophylaxis

**Published:** 1921-06

**Authors:** Julio Endelman


### DISCRIMINATING PROPHYLAXIS

Of late considerable discussion has arisen concerning the inclusion in the child's diet of some fruit, such as oranges, apples or grapes. It is a generally accepted fact that the stimulation of salivary secretion, in both quantity and alkalinity, can be brought about by the presence in the mouth of acid foods such as the fruits above mentioned. An acid fruit, the orange for instance, will stimulate a flow of saliva of greater alkalinity than resting saliva, the alkalinity persisting for quite some time after the acid fruit is ingested. On the other hand, starchy foods and sweets depress both the amount and the alkalinity of the saliva. Therefore, bread and butter, one of the most widely consumed articles of food by children, should be followed in the dietary by an orange or apple. In bread from sixty to eighty per cent is starch. In the process of mouth digestion, the starch through the agency of ptyalin or salivary amylose is converted into maltose, a double sugar, $C_{12}H_{22}O_{11}$. If any of these bread particles become adherent to the surfaces of the enamel, and saliva is secreted in insufficient amounts because of the bad masticatary habits of the child, no fluid will be available to wash them off, and caries will soon follow. It is, of course, admitted that the hard foods must be rendered soft before deglutition can take place, but in the accomplishment of this phase of digestion through thorough mastication, a flow of saliva is produced, greatly exceeding indifferent or insufficient mastication. The adherence of soft food particles takes place in preference upon enamel surfaces presenting rough or uneven areas. Prophylactic measures in the child should embrace the detection of such rough surfaces and their correction, by polishing with hard wood points on the hand polisher and finely powdered pumice, or if this technique be insufficient with stones of proper grit followed by the application of pumice, precipitated chalk, tin oxid and chalk or any other powder or combination that will produce a high luster. A survey of the etiology of dental caries brings out the fact, confirmed by clinical observation, that individuals up to the age of about twenty-one are more susceptible to the inroads of decay than they are in later years, when in most people periods of immunity to the disease seem to be the case. Before determining what degree of grinding and polishing should be applied to enamel surfaces, we should consider the patient's age, and his high, medium or low susceptibility to caries. In patients who have attained the age of relative immunity to caries no grinding should be resorted to, especially of such teeth as exhibit no caries at all. In such teeth the enamel is sufficiently resistant to the inroad of caries. A ground enamel surface to which subsequently a high luster has been given is better as a medium of defense than an enamel surface presenting congenital defects such as pits or grooves. But an enamel surface free from congenital irregularities is also better than the most highly artificially polished enamel. The degree of permeability to the acid end products of carbonhydrate fermentation is greater in enamel surfaces that have been ground than in those which have been permitted to retain their natural polish. Indiscriminate enamel grinding as a prophylactic measure of caries is to be condemned; it is a dangerous procedure; it is unscientific; and hence rather harm than benefit must accrue to the patient.—Julio Endelman, in *Pacific Gazette.*

